# Proximate composition of wild meats present in traditional food systems of the Brazilian Amazon

**DOI:** 10.1371/journal.pone.0327783

**Published:** 2025-07-21

**Authors:** Thales Daniel Oliveira de Lima e Silva, Andrezza Cavalcante Nunes Matias, Elias Jacob de Menezes Neto, João Valsecchi do Amaral, Lorena Ianka Pontes da Silva, Michelle Cristine Medeiros Jacob, Juliana Kelly da Silva-Maia, Daniel Tregidgo

**Affiliations:** 1 Federal University of Rio Grande do Norte, Natal, Rio Grande do Norte, Brazil; 2 Mamirauá Institute for Sustainable Development, Tefé, Amazonas, Brazil; State University of Bangladesh, BANGLADESH

## Abstract

The Amazon region, renowned for its rich biodiversity, is home to indigenous and riverside populations that maintain an intrinsic relationship with the forest, playing a crucial role in the local ecological balance. The diet of this population, based on fish and wild meat, is essential for their food security, providing proteins and other fundamental nutrients. However, the nutritional composition of these meats remains under-researched, limiting the development of appropriate nutritional strategies and interventions. This study aimed to characterize the proximate composition of mammal, bird, and reptile wild meat consumed within the context of a traditional food system in the Brazilian Amazon. A total of 78 samples were collected, including 51 mammals, 18 birds, and 9 reptile samples, from the Tefé National Forest, and the Amanã and Mamirauá Sustainable Development Reserves. The muscle tissues of the samples were analyzed following the official methods of the Association of Official Analytical Collaboration. Differences between taxonomic classes were evaluated using the Kruskal-Wallis test, followed by Dunn’s test with Bonferroni adjustment (P < 0.05) for those species with three or more replicates. Across all samples, descriptive statistics showed that the mean protein contents were 22.27 (1,32), 23.47 (1.7), and 22.31 (1.3) g/100 g of meat for mammals, birds, and reptiles, respectively. The mean ash contents were 1.08 (0.11), 1.27 (0.1), and 1.08 (0.13) g/100 g of meat, while the lipid contents were 5.72 (1,72), 5.40 (1.58), and 4.03 (0.53) g/100 g of meat for the same groups. Additionally, results indicated that macronutrient and ash (p = 0.13) levels did not differ significantly among classes: lipids (p = 0.07164) and proteins (p = 0.3055). When comparing the meats of wild mammals, birds, and reptiles with domestic meats typically consumed in modern food systems, a nutritional similarity was observed, highlighting their importance as key nutrient sources, particularly proteins. The data obtained provide a foundation for further research and will serve as a basis for strategies aimed at promoting food and nutritional security, contributing to the reduction of maternal and child malnutrition in Amazonian communities. Furthermore, the novel quantification of the proximate composition of wild meats underscores their role as a strategic resource for promoting health in these populations.

## Introduction

The United Nations (UN) has established a global commitment among various countries known as the Sustainable Development Goals (SDGs), which define targets for reducing inequalities, including eradicating poverty and hunger in developing, poor, and vulnerable populations [1]. Among these vulnerable populations are traditional rural Amazonian communities, namely indigenous peoples, river-dwelling *ribeirinhos*, and Afro-descendent *quilombolas*. Brazilian research demonstrates that this region suffers the country’s worst food security [2,3], and that rural Amazonian *ribeirinho* and indigenous childhood anemia rates of approximately two-thirds [4,5] are around six-times higher than the national average [6].

Social inequalities and geographical remoteness shape the vulnerability of much of the Amazonian population, while the annual floods can significantly exacerbate food insecurity in many rural areas, as farmland is inundated and fishing becomes more difficult [7–9]. Rivers play a fundamental role in the culture and lives of riverine communities, serving not only as a source of food but also as important routes for transportation and trade [10]. The dynamic nature of hydrological cycles shapes the life and subsistence of Amazonian communities, playing a crucial role in their cultures and way of life [11]. The geographic isolation and the absence or poor quality of basic public services (such as water, sanitation, health, and education) shape the vulnerability faced by the Amazonian population, while the hydrological cycle of floods and droughts plays a crucial role in their culture and way of life.

Amazonian peoples have long benefited from these natural cycles, which influence food access through plant gathering and animal sources (fishing and hunting), as well as agricultural and livestock production [12,13]. While the dry season generally allows for increased fishing and agricultural production, the flood season ensures the high productivity of flooded environments, enhancing access to forest products and wild meat. However, it is also during the flood season that food insecurity can worsen in many rural areas, as agricultural lands become submerged and fishing becomes more difficult [7–9].

This dynamic is natural, but it has been increasingly affected by climate change. Extreme events of flooding and drought have isolated Amazonian communities more frequently: schools are closed, health services are suspended, fishing and hunting activities are halted, and agricultural production is severely disrupted [14].

Communities maintain a close relationship with their surrounding environment and dependence on natural resources for their food and livelihood, including fishing, and the extraction of forest products [11,15,16]. However urban influences are increasing, and an ongoing nutrition transition from wild and locally cultivated foods towards industrialised products is widespread across the Amazon region [17,18]. Fishing and hunting remain essential practices both culturally and for food and nutritional security [19]. Dietary research shows a reliance on fish and manioc (*Manihot esculenta* Crantz) among Amazonian communities, with local fruits and wildmeat playing a central important role in certain regions and times, particularly in contexts of fish scarcity [2,20].

The consumption of wild meat occurs year-round. However, during the flood season, when river levels in Central Amazonia can rise by up to 15 meters, there is an increase in the consumption of wild species such as paca (*Cuniculus paca*), tapir (*Tapirus terrestris*), white-lipped peccary (*Tayassu pecar*i), and agouti (*Dasyprocta* spp.). This shift is attributed to the difficulty in fishing during this period due to the dispersal of fish across flooded rivers and forests, reducing catch rates [4,9,21], but also to the ecological and behavioral characteristics of the species and the hunting practices of traditional peoples [22]. The consumption of wild meat by traditional peoples is a significant cultural identity marker among these social groups, making it essential to respect their dietary traditions [23].

The importance of wild meat for the nutrition and health of riverine populations has gained increasing recognition. A recent study demonstrated a positive relationship between wild meat consumption and hemoglobin levels in riverine children from rural areas, a group particularly vulnerable to iron-deficiency anemia [4]. Additionally, wild meat, along with fish, contributes to the protein intake of this population, as protein deficiencies and other essential nutrient inadequacies still lead to cases of malnutrition in these communities [20].

Wild meat consumption can serve as an important strategy to promote food security in riverine communities and other vulnerable populations. In the Amazon, particularly in rural areas, persistent levels of food insecurity and maternal-child malnutrition underscore the need for focused attention on these populations, which are characterized by traditional food systems [24,25]. Protein deficiencies in mothers, for example, increase the risk of childhood malnutrition, perpetuating the cycle of poverty [26]. However, the lack of precise data on the nutritional composition of wild meat hinders the development of scientifically grounded strategies to address these nutritional deficiencies [27,28].

The assertion that wild meat can contribute to the food security of Amazonian communities is primarily based on approximations to the nutritional profiles of domesticated animal meat [29]. However, such extrapolations may be limited. A recent study revealed that wild bird meat may have higher iron levels than red mammal meat, possibly due to behavioral differences and slaughtering practices, challenging the common perception in nutrition that mammal meat contains higher iron levels [30].

Thus, while nutritional parallels between wild and domesticated meat are frequently drawn, these comparisons lack robust scientific evidence, primarily due to the scarcity of precise data on the nutritional profiles of wild meat. Currently available food composition tables have significant limitations in characterizing the nutritional content of wild animal meat, resulting in a knowledge gap regarding a crucial component of traditional diets [31]. Additionally, tools such as the United Nations Food Matching database, which facilitates nutritional composition estimations when data are unavailable, could be more reliably utilized if more data on wild meat composition were available [32].

An important gap to be addressed is understanding how nutritional composition varies across different taxonomic classes of wild animals. Preliminary studies suggest significant differences in micronutrient content between various animal classes [30]. However, little is known about variations in macronutrient content (proteins, lipids) in these animals. Our hypothesis, based on the behavior of macronutrients in domesticated animal meat [33], is that variations among different animal classes are minimal. Nutritional characterization using primary data will enable us to generate robust findings that can support actions aimed at promoting food and nutritional security for these populations.

Therefore, this study aims to characterize the proximate composition of wild mammals, birds, and reptiles consumed in traditional food systems in the Brazilian Amazon, specifically in the Middle Solimões region.

## Materials and methods

### Ethics and permission

The study obtained the necessary authorization to conduct scientific activities (SISBIO, no. 77176−2) for sampling animals, as well as prior approval for working in the conservation units from relevant Brazilian Federal (ICMBio: Ministry of Environment’s Chico Mendes Institute for Biodiversity Conservation) and Amazonas State (SEMA: Department of the Environment) authorities. Additionally, the study was approved by the Mamirauá Sustainable Development Institute’s Research Ethics Committee (CEP) (CAAE: 55899222.7.0000.8117) and Animal Use Ethics Committee (CEUA) (Protocol nº02/2022). Permission to collect samples was initially granted by community leaders, followed by the individual community members that donated samples, whose identities were never noted, to maintain anonymity.

### Sampling

Opportunistic sampling was used to collect samples of wild meat (muscle tissue), based on donations from the community members, respecting the traditional subsistence practices of the local communities, without promoting hunting beyond the usual needs of the residents. The 78 samples, distributed across 20 different species, were collected between 2022 and 2024 in three distinct protected areas in the Central Brazilian Amazon: Amanã Sustainable Development Reserve (RDS), Mamirauá Sustainable Development Reserve (RDS), and the Tefé National Forest (FLONA) (Table 1).

**Table 1:** Popular and scientific name of the species present in the sample.

Popular name	Scientific name	Family	Class	Number of samples
Anta	*Tapirus terrestris* Linnaeus	*Tapiridae*	Mammals	1
Biguá	*Phalacrocorax brasilianus* Gmelin	*Phalacrocoracidae*	Birds	1
Catitu	*Pecari tajacu* Linnaeus	*Tayassuidae*	Mammals	1
Cujubim	*Pipile cumanensis* Jacquin	*Cracidae*	Birds	1
Cutia	*Dasyprocta fuliginosa* Wagler	*Dasyproctidae*	Mammals	9
Jacaré-Açu	*Melanosuchus niger* Spix	*Alligatoridae*	Reptiles	3
Jacaré-paguá	*Paleosuchus palpebrosus* Cuvier	*Alligatoridae*	Reptiles	1
Jacaretinga	*Caiman crocodilus* Linnaeus	*Alligatoridae*	Reptiles	4
Jacú	*Penelope jacquacu* Spix	*Cracidae*	Birds	5
Macaco guariba	*Alouatta juara*	*Atelidae*	Mammals	3
Macaco Prego	*Sapajus apella* Linnaeus	*Cebidae*	Mammals	1
Maguari	*Ardea cocoi* Linnaeus	*Ardeidae*	Birds	1
Mergulhão	*Mergus octosetaceus* Vieillot	*Anatidae*	Birds	1
Mutum-cavalo	*Mitu tuberosum* Spix	*Cracidae*	Birds	2
Mutum-piuri	*Crax globulosa* Spix	*Cracidae*	Birds	3
Paca	*Cuniculus paca* Linnaeus	*Cuniculidae*	Mammals	27
Pato do mato	*Cairina moschata* Linnaeus	*Anatidae*	Birds	4
Perema	*Mesoclemmys raniceps* Gray	*Chelidae*	Reptiles	1
Tatupeba	*Dasypus novemcinctus* Linnaeus	*Dasypodidae*	Mammals	4
Veado Capoeira	*Mazama americana* Erxleben	*Cervidae*	Mammals	5

Samples of raw muscle tissue (approximately 50 g) were obtained using ceramic knives, which were sanitized between collections with detergent, water, and 70% ethanol to avoid cross-contamination. The choice of muscle tissue is justified by its status as the most commonly consumed part among the riparian communities. Following field collection, the samples were immediately frozen in liquid nitrogen and subsequently stored in a freezer at −18°C. The samples were then lyophilized (freeze-dried) at the Mamirauá Institute, resulting in approximately 10 g of material, a process aimed at preserving the samples and facilitating transport to the Federal University of Rio Grande do Norte (UFRN).

### Moisture

The determination of moisture content was conducted at the Mamirauá Institute using raw samples, following method 425.45b from the Association of Official Analytical Chemists (AOAC) [34]. The procedure consisted of the following steps: initially, Petri dishes and porcelain crucibles were dried in an oven at 105°C for 2 hours. Then, the glassware was transferred to a desiccator for approximately 30 minutes until it reached ambient temperature. After weighing on an analytical balance, aliquots of 2 g of the sample were added in triplicate. The samples were then subjected to drying in an oven at 105°C until reaching a constant weight or overnight. After cooling in the desiccator, a final weighing was performed, and the results were expressed as a percentage. To complement the database, missing moisture values were imputed, resulting in an average percentage error of 5%.

### Imputation of missing moisture data

To obtain a complete dataset for analysis, we imputed missing values for moisture content. This was required due to limited sample mass obtained from some donated samples [35], meaning that some samples had only proximate composition data determined from lyophilized tissue, thus lacking raw moisture measurements. The imputation process was framed as a multivariate regression problem, predicting the missing moisture values for a sample based on its other measured nutritional components.

While the missing data was related to external constraints, it is reasonable to assume that they were Missing Completely at Random (MCAR) or Missing at Random (MAR) relative to the measured nutritional variables, as the cause of missingness (sampling limitations) was independent of the underlying true moisture values.

We evaluated several machine learning techniques using cross-validation to select the most suitable approach. The methods included Multiple Imputation by Chained Equations (MICE), K-Nearest Neighbors (KNN), Soft Imputation, Matrix Factorization, Nuclear Norm Minimization, and Iterative Singular Value Decomposition (SVD). All methods were assessed using the Symmetric Mean Absolute Percentage Error (SMAPE) and the Mean Absolute Error (MAE). The technique yielding the lowest SMAPE (7.24%) was selected for the final imputation, which indicated the best balance of precision and consistency across the predictions. We performed all imputation analyses using Python.

### Ash content

The determination of fixed mineral residues was performed according to method 923.03 of the AOAC [34], which recommends incineration in a muffle furnace at 550°C. To optimize the use of the samples, given the limited quantity remaining after lyophilization, pilot tests were conducted beforehand. In these tests, aliquots of approximately 0.5 g in triplicate showed similar results to those obtained with the AOAC method (which uses 2–5 g in triplicate), validating the adoption of 0.5 g for this study. Thus, aliquots of 0.5 g of lyophilized sample were weighed into porcelain crucibles previously dried in a muffle furnace at 105°C for 2 hours, cooled in a desiccator for 20 minutes, and weighed. The samples were then subjected to carbonization on a heated plate until smoke emission ceased, after which they were transferred to the muffle furnace. The calcination was performed at 550°C until light ashes were obtained (approximately 12 hours). The muffle furnace temperature was gradually increased in increments of approximately 100°C until stabilization at 550°C was reached. For weighing, after cooling in the desiccator for 20 minutes, the samples were weighed and returned to the muffle furnace for an additional one hour. This weighing cycle was repeated two to three times, depending on the stabilization of the mass. The results were expressed as a percentage of dry sample and subsequently converted to wet basis.

### Protein content

The protein content was determined based on nitrogen content, according to the micro-Kjeldahl method 960.52 of the AOAC [34]. The procedure consists of three stages: digestion, distillation, and titration. First, 150 mg of the lyophilized sample were weighed onto filter paper and transferred to Micro-Kjeldahl tubes with 2 g of catalytic mixture (10:1 ratio of sodium sulfate and copper sulfate) and 5 mL of sulfuric acid. The tubes were heated in a digestion block to a temperature of 450°C, with a progressive temperature increase of 100°C until stabilization. The digestion process lasted 12 hours, counted after the stabilization of the equipment. For solubilization before the distillation process, 10 mL of distilled water was used on a vortex shaker until the complete dissolution of the contents. In the distillation process, the tubes with digested samples were connected to a protein distiller and received 20 mL of 30% sodium hydroxide. During sample exchanges, the equipment was cleaned with distilled water to avoid cross-contamination. At the outlet of the distiller (tip of the condenser), a 10 mL Erlenmeyer flask containing 3% boric acid solution and 4 drops of 2% mixed indicator (Methyl Red + Methylene Blue) was used to collect the distilled ammonia until the approximate volume of 100 mL. Finally, titration was performed with 0.1 molar hydrochloric acid solution. The percentage of protein in the lyophilized sample was calculated using the following equation: Proteins (% m/m) = V × 0.14 × f/ P, where V = volume of sulfuric acid used in titration, P = grams used in the titration, and f = the nitrogen conversion factor of 6.25 expressed on a dry basis and then converted to a wet basis. All samples were analyzed in triplicate

### Lipid content

The determination of ether extract content was performed by extraction with ethyl ether using a Soxhlet extractor. The Soxhlet method 920.39 [34] is based on the repeated action of the organic solvent on the sample, promoting the complete removal of fat through solubilization. The volatile solvent is subsequently eliminated, allowing for the determination of the lipid fraction. To optimize the use of the small quantities of samples remaining from lyophilization, pilot tests were conducted prior to the experiment. In these tests, aliquots of approximately 0.5 g of sample in triplicate showed results similar to those reported by the AOAC method (which recommends 3–5 g in triplicate), validating the use of 0.5 g in the present study. Aliquots of 0.5 g of the sample were placed in filter paper cartridges, which were positioned in appropriate supports in the Soxhlet extractor. A volume of 80 mL of ethyl ether was transferred to the Soxhlet flask, previously tared (heated at 105°C for two hours, cooled in a desiccator for 20 minutes, and weighed). The cartridge containing the sample was submerged in the ether and heated under reflux for five hours. Then, the cartridge was moved to the intermediate region of the apparatus for an additional hour. After completing six hours of extraction, the condenser was closed, and the solvent was collected for approximately 30 minutes. The flask was then dried in an oven at 105°C for one hour, cooled in a desiccator for 20 minutes, weighed, and the ether extract content was determined by the difference in mass. The lipid content results will be expressed as a percentage of dry matter and converted to wet basis.

### Total carbohydrate

For the calculation of total energy value, the total carbohydrate content in the lyophilized samples was considered, obtained by difference calculation according to the formula [36].


Total\ Carbohydrates = 100 − Moisture\+\Ashes\+\Lipids\+\Protein

### Total energy value (kJ/kcal)

The total caloric value was calculated according to Atwater’s conversion factors: 9 kcal/g for lipids and 4 kcal/g for carbohydrates and proteins, and then multiplied by 4.184 for presentation in kJ [36].

### Comparison of the nutritional composition among classes

For the comparisons between taxonomic classes, only species with three or more samples per animal species were included. This approach aimed to strengthen scientific rigor and avoid the emergence of biases that could affect the results. The species analyzed were: Spix’s Guan (*Penelope jacquacu*), Wattled Curassow (*Crax globulosa*), Muscovy Duck (*Cairina moschata*), Black Agouti (*Dasyprocta fuliginosa*), Howler Monkey (*Alouatta juara*), Lowland Paca (*Cuniculus paca*), Six-banded Armadillo (*Dasypus novemcinctus*), Red Brocket Deer (*Mazama americana*), Black Caiman (*Melanosuchus niger*), and Spectacled Caiman (*Caiman crocodilus*).

### Comparison of the nutritional composition of wild meats and commonly consumed meats

The composition data were stratified by taxonomic class: birds, mammals, and reptiles. Only species with three or more samples were included, and compared qualitatively with the nutritional composition of commonly consumed domestic meats: chicken, beef, and pork (*Gallus gallus*, *Bos taurus*, and *Sus spp*., respectively). The comparison of the composition of meats traditionally consumed by the Brazilian population with the data from the present study was made against the information from the Brazilian Food Composition Table (TBCA) [37]. To approximate the comparison of the meats with the data obtained by opportunistic sampling (donations without prior specification of the exact meat cuts), the average of various cuts of beef, chicken, and pork directly obtained from the TBCA was used (Table 2).

**Table 2:** Description and codes of commonly consumed meats.

Code	Description
BRC0118F	Meat, chicken, raw (average of several cuts), Brazil
BRC0154F	Meat, pork, raw (average of several cuts), Brazil
BRC0049F	Meat, beef, forequarter, raw (average of several cuts), Brazil

Data presented in the Brazilian Food Composition Table (TBCA) used for comparison.

### Protein contribution per portion of wild meat for adults and children

The percentage contributions from consuming wild meats were determined based on the Recommended Dietary Allowances (RDA) and Estimated Average Requirements (EAR) for the life stages of adult women and children [38]. Only species with three or more samples were included. For the EAR, values were converted to the average weight for each age group to obtain the recommended amounts in grams per day. The portion sizes for adults were based on the household measures proposed by the TBCA, while for children, the recommendations from the Dietary Guidelines for Brazilian Children Under two Years of Age were used [39].

### Statistical analysis

All analyses were performed in triplicate, with results expressed as mean ± standard deviation. Descriptive statistics were provided. For species with three or more replicates, statistical analysis was conducted to compare the nutritional composition between classes. Prior to this, the obtained results were evaluated for normality (Shapiro-Wilk test). Non-parametric data were evaluated using the Kruskal-Wallis test followed by Dunn’s test with Bonferroni adjustment, with P < 0.05 considered significant. The null hypothesis assumed that there was no significant difference between the medians of the compared groups (class), and the alternative hypothesis assumed that at least one group had a significantly different median compared to the others. Statistical analysis was performed using the R programming language through the RStudio interface (version 4.4.2).

### Inclusivity in global research

Additional information regarding the ethical, cultural, and scientific considerations specific to inclusivity in global research is included in the Supporting Information (S1 Checklist).

## Results

### Proximate composition

The protein levels were considerable in all species analyzed, the average protein content found was 22.27, 23.47, and 22.31 g/100g of meat in mammals, birds, and reptiles, respectively. In mammals, the average was 22.27 g/100 g of meat, with the red brocket deer (*Mazama americana*) showing the highest content (23.19 g/100 g). Birds presented protein levels ranging from 17.86 to 29.61 g/100 g of meat, with an average of 23.47 g/100 g, with the Razor-billed curassow (*Mitu tuberosum*) standing out at 25.05 g/100 g. In reptiles, the levels ranged from 19.02 to 24.71 g/100 g of food, with an average of 22.31 g/100 g of meat, and the Cuvier’s dwarf caiman (*Paleosuchus palpebrosus*) showed the highest concentration, with 23.96 g/100 g (Table 3).

**Table 3:** Average values of nutrients in wild meat from different classes.

Species	Moisture	Protein (g/100 g)	Lipid (g/100 g)	Ash (g/100 g)
**Mammals (n = 51)**	**73.84 ± 1.13** **(72.62 - 76.06)**	**22.27 ± 1.32** **(13.27 - 30.35)**	**5.72 ± 1.72** **(1.81 - 17.34)**	**1.08 ± 0.11** **(0.56 - 1.23)**
*Cuniculus paca (n = 27)*	73.45 ± 1.87 (69.16 - 76.70)	22.09 ± 3.06 (13.27 - 30.35)	5.97 ± 2.96 (1.81 - 17.34)	1.17 ± 0.17 (0.73 - 1.47)
*Dasyprocta fuliginosa (n = 9)*	73.82 ± 1.87 (72.87 - 76.09)	22.71 ± 1.40 (20.75 - 25.17)	4.07 ± 1.03 (2.56 - 5.63)	1.23 ± 0.14 (0.92 - 1.49)
*Mazama americana (n = 5)*	72.81 ± 0.70 (72.21 - 73.93)	23.19 ± 1.41 (21.61 - 24.88)	4.84 ± 1.01 (4.07 - 6.45)	1.02 ± 0.28 (0.56 - 1.23)
*Dasypus novemcinctus (n = 4)*	73.31 ± 1.23 (72.38 - 75.03)	22.30 ± 3.50 (18.66 - 26.27)	7.53 ± 4.40 (3.32 - 13.22)	1.10 ± 0.17 (0.94 - 1.26)
*Alouatta juara (n = 3)*	74.85 ± 0.40 (73.81 - 74.54)	23.05 ± 1.01 (22.21 - 24.17)	4.51 ± 1.71 (2.69 - 6.10)	1.03 ± 0.13 (0.89 - 1.14)
*Tapirus terrestris (n = 1)*	72.62 ± 0.00 (72.62)	21.73 ± 0.00 (21.73)	8.98 ± 0.00 (8.98)	0.94 ± 0.00 (0.94)
*Sapajus apella(n = 1)*	73.81 ± 0.00 (73.81)	23.7 ± 0.00 (23.7)	5.38 ± 0.00 (5.38)	1.22 ± 0.00 (1.22)
*Pecari tajacu (n = 1)*	76.06 ± 0.00 (76.06)	19.42 ± 0.00 (19.42)	4.44 ± 0.00 (4.44)	0.97 ± 0.00 (0.97)
**Birds (n = 18)**	**72.18 ± 1.08** **(70.02 - 73.52)**	**23.47 ± 1.7** **(17.86 - 29.61)**	**5.40 ± 1.58** **(0.00 - 9.26)**	**1.27 ± 0.1** **(0.97 - 1.49)**
*Phalacrocorax brasilianus (n = 1)*	72.19 ± 0.00 (72.19)	21.65 ± 0.00 (21.65)	7.87 ± 0.00 (7.87)	1.18 ± 0.00 (1.18)
*Pipile cumanensis (n = 1)*	71.63 ± 0.00 (71.63)	24.99 ± 0.00 (24.99)	4.56 ± 0.00 (4.56)	1.39 ± 0.00 (1.39)
*Ardea cocoi (n = 1)*	72.52 ± 0.00 (72.52)	22.06 ± 0.00 (22.06)	4.17 ± 0.00 (4.17)	1.23 ± 0.00 (1.23)
*Penelope jacquacu (n = 5)*	71.79 ± 1.33 (69.58 - 72.99)	24.78 ± 2.91 (22.22 - 29.61)	5.53 ± 0.46 (4.88 - 6.19)	1.34 ± 0.10 (1.20 - 1.49)
*Mergus octosetaceus (n = 1)*	70.02 ± 0.00 (70.02)	25.08 ± 0.00 (25.08)	4.27 ± 0.00 (4.27)	1.4 ± 0.00 (1.4)
*Mitu tuberosum (n = 2)*	72.70 ± 0.24 (72.52 - 72.87)	25.05 ± 2.35 (23.39 - 26.72)	3.53 ± 0.64 (3.08 - 3.99)	1.26 ± 0.01 (1.25 - 1.27)
*Crax globulosa (n = 3)*	73.07 ± 0.95 (72.52 - 74.18)	21.10 ± 2.80 (17.86 - 22.78)	5.87 ± 3.45 (0.00 - 6.51)	1.18 ± 0.27 (0.97 - 1.49)
*Cairina moschata (n = 4)*	73.52 ± 0.99 (72.39 - 74.65)	23.06 ± 2.09 (20.81 - 25.46)	7.42 ± 1.33 (6.38 - 9.26)	1.17 ± 0.05 (1.11 - 1.24)
**Reptiles (n = 9)**	**74.28 ± 1.84** **(72.48 - 75.96)**	**22.31 ± 1.3** **(19.02 - 24.71)**	**4.03 ± 0.53** **(0.00 - 5.74)**	**1.08 ± 0.13** **(0.96 - 1.29)**
*Melanosuchus niger* (n = 3)	75.76 ± 2.68 (72.76 - 77.95)	22.64 ± 1.47 (21.51 - 24.31)	4.69 ± 0.71 (4.23 - 5.51)	1.08 ± 0.12 (0.96 - 1.20)
*Paleosuchus palpebrosus* (n = 1)	72.48 ± 0.00 (72.48)	23.96 ± 0.00 (23.96)	3.67 ± 0.00 (3.67)	1.26 ± 0.00 (1.26)
*Caiman crocodilus* (n = 4)	75.96 ± 2.10 (72.84 - 77.25)	20.97 ± 1.88 (19.02 - 23.49)	3.55 ± 2.49 (0.00 - 5.74)	0.98 ± 0.13 (0.98 - 1.29)
*Mesoclemmys ranicep*s (n = 1)	72.90 ± 0.00 (72.90)	21.67 ± 0.00 (21.67)	4.21 ± 0.00 (4.21)	0.99 ± 0.00 (0.99)

Expressed on a wet basis and presented with standard deviation and distribution range.

Regarding lipids, mammals presented concentrations ranging from 1.81 to 17.34 g/100 g of meat, with an average of 5.72 g/100 g per portion. The species with the highest lipid levels were the lowland tapir (*Tapirus terrestris*) with 8.89 g/100 g of meat. In birds, the values ranged from 0.00 to 9.26 g/100 g, with an average of 5.40 g/100 g of meat, with the neotropical cormorant (*Phalacrocorax brasilianus*) standing out at 7.87 g/100 g. In reptiles, concentrations ranged from 0.00 to 5.74 g/100 g of meat, with an average of 4.03 g/100 g of meat. The black caiman (*Melanosuchus niger*) showed the highest lipid level in the class, with 4.69 g/100 g, demonstrating relevant levels of this macronutrient.

Regarding moisture content, mammals ranged from 72.62 to 76.06% per 100 g of meat, with the white-lipped peccary (*Pecari tajacu*) standing out at 76.06%. In birds, moisture content varied from 70.02 to 73.52% per 100 g of meat, with the highest average found in the muscovy duck (*Cairina moschata*) at 73.52% per 100 g of food. Finally, in reptiles, moisture content ranged from 72.48 to 75.69% per 100 g, with the spectacled caiman (*Caiman crocodilus*) standing out at 75.96% per 100 g of meat.

Lastly, the ash content in mammals ranged from 0.56 to 1,23 g/100 g of meat, with an average of 1.08 g/100 g. The black agouti (*Dasyprocta fuliginosa*) had the highest ash content in this group (1.23 g/100 g of meat). In birds, values ranged from 0.97 to 1.49 g/100 g of meat, with an average of 1.27 g/100 g, and the highest average value was found in the piping guan (*Pipile cumanensis*), with 1.39 g/100 g. Reptiles had the lowest ash concentrations among the groups, ranging from 0.96 to 1.29 g/100 g of meat, with an average of 1.08 g/100 g per portion. The black caiman (*Melanosuchus niger*) was the highest in this group, with 1.26 g/100 g.

The total caloric values of wild meats ranged from the lowest value of 121.32 kJ (507.60 Kcal) in the white-lipped peccary (*Pecari tajacu*) to the highest value of 184,82 kJ (773,29 Kcal) in the lowland tapir tapir (*Tapirus terrestris)* (Table 4).

**Table 4:** Proximate Composition of wild meat.

Popular name	Species	Energy (kJ)	Energy (kcal)	Moisture (g)	Total carbohydrate (g)	Protein (g)	Lipid (g)	Ash (g)
Paca	*Cuniculus paca (n = 27)*	639.34	152.81	73.45 ± 1.87	2.68	22.09 ± 3.06	5.97 ± 2.96	1.17 ± 0.17
Cutia	*Dasyprocta fuliginosa (n = 9)*	563.96	134.79	73.82 ± 1.87	1.83	22.71 ± 1.40	4.07 ± 1.03	1.23 ± 0.14
Veado Capoeira	*Mazama americana (n = 5)*	601.42	143.74	72.81 ± 0.70	1.86	23.19 ± 1.41	4.84 ± 1.01	1.02 ± 0.28
Tatupeba	*Dasypus novemcinctus (n = 4)*	727.64	173.91	73.31 ± 1.23	4.24	22.30 ± 3.50	7.53 ± 4.40	1.10 ± 0.17
Macaco guariba	*Alouatta juara (n = 3)*	613.17	146.55	74.85 ± 0.40	3.44	23.05 ± 1.01	4.51 ± 1.71	1.03 ± 0.13
Anta	*Tapirus terrestris (n = 1)*	773.29	184.82	72.62 ± 0.00	4.27	21.73 ± 0.00	8.98 ± 0.00	0.94 ± 0.00
Macaco Prego	*Sapajus apella(n = 1)*	668.02	159.66	73.81 ± 0.00	4.11	23.70 ± 0.00	5.38 ± 0.00	1.22 ± 0.00
Catitu	*Pecari tajacu (n = 1)*	507.60	121.32	76.09 ± 0.00	0.92	19.42 ± 0.00	4.44 ± 0.00	0.97 ± 0.00
Biguá	*Phalacrocorax brasilianus (n = 1)*	707.05	168.99	72.19 ± 0.00	2.89	21.65 ± 0.00	7.87 ± 0.00	1.18 ± 0.00
Cujubim	*Pipile cumanensis (n = 1)*	632.96	151.28	71.63 ± 0.00	2.57	24.99 ± 0.00	4.56 ± 0.00	1.39 ± 0.00
Maguari	*Ardea cocoi (n = 1)*	525.89	125.69	72.52 ± 0.00	0.00	22.06 ± 0.00	4.17 ± 0.00	1.23 ± 0.00
Jacú	*Penelope jacquacu (n = 5)*	680.46	162.63	71.79 ± 1.33	3.44	24.78 ± 2.91	5.53 ± 0.46	1.34 ± 0.10
Mergulhão	*Mergus octosetaceus (n = 1)*	593.42	141.83	70.02 ± 0.00	0.77	25.08 ± 0.00	4.27 ± 0.00	1.40 ± 0.00
Mutum-cavalo	*Mitu tuberosum (n = 2)*	594.59	142.11	72.70 ± 0.24	2.54	25.05 ± 2.35	3.53 ± 0.64	1.26 ± 0.01
Mutum-piuri	*Crax globulosa (n = 3)*	594.64	142.12	73.07 ± 0.95	1.22	21.10 ± 2.80	5.87 ± 3.45	1.18 ± 0.27
Pato do mato	*Cairina moschata (n = 4)*	751.83	179.69	73.52 ± 0.99	5.17	23.06 ± 2.09	7.42 ± 1.33	1.17 ± 0.05
Jacaré-Açu	*Melanosuchus niger (n = 3)*	555.51	132.77	75.76 ± 2.68	4.17	22.64 ± 1.47	4.69 ± 0.71	1.08 ± 0.12
Jacaré-paguá	*Paleosuchus palpebrosus (n = 1)*	562.12	134.35	72.48 ± 0.00	1.37	23.96 ± 0.00	3.67 ± 0.00	1.26 ± 0.00
Jacaretinga	*Caiman crocodilus (n = 4)*	484.63	115.83	75.96 ± 2.10	1.46	20.97 ± 1.88	3.55 ± 2.49 0	0.98 ± 0.13
Perema	*Mesoclemmys raniceps (n = 1)*	521.20	124.57	72.90 ± 0.00	0.00	21.67 ± 0.00	4.21 ± 0.00	0.99 ± 0.00

### Comparison between classes

**Fig 1:**
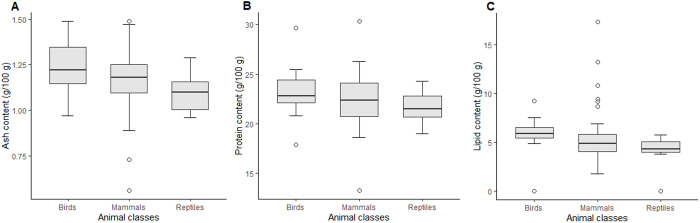
Comparison of protein, ash, and lipid contents between animal classes.

The results indicated that protein, lipid and ashes levels did not differ significantly between classes: Proteins (p = 0.3055), ashes (p = 0.13) and lipids (p = 0.07164) (Fig 1). The results of the statistical analysis are presented in Supplementary Material 1 (S2 File).

### Comparison of the nutritional composition of wild meats and commonly consumed meats

The meats from mammals, birds, and reptiles can be compared with the domestic meats commonly consumed by the Brazilian population, as they are considered an excellent nutritional source (Table 5).

**Table 5:** Comparison of the Nutritional Composition of Wild Meats and Commonly Consumed Meats.

Species/Class	Protein (g/100g)	Ash (g/100g)	Lipid (g/100g)
Beef Bos taurus* (BRC0049F)	19.7	0.98	7.79
Chicken meat Gallus gallus* (BRC0118F)	18.7	0.93	8.21
Pork Suss spp* (BRC0154F)	19.8	1.11	11.1
Wild-Mammals**	22.27 (13.27–30.35)	1.08 (0.56–1.23)	5.72 (1.81–17.34)
Wild-Birds**	23.47 (17.86–29.61)	1.27 (0.97 −1.49)	5.40 (0.00–9.26)
Wild-Reptiles**	22.31 (19.02–24.31)	1.08 (0.96–1.29)	4.03 (0.00–5.74)

Source: TBCA. *Means of various cuts compiled by TBCA. **Class means obtained in the study. (Range of means).

### Protein contribution per portion of wild meat for adults and children

Wild meats demonstrated an important contribution to the diet of women and children, as all groups contributed at least half of the RDA and EAR protein recommendations for both adult women and children aged 1–8 years (Table 6), considering the consumption of one portion per day.

**Table 6:** Protein Contribution per Portion of Wild Meat at Different Life Stages.

Life stage	Portion (g)	RDA (g/day)	EAR (g/day)	% Contribution of mammals (RDA/EAR)	% Contribution of birds (RDA/EAR)	% Contribution of reptiles (RDA/EAR)
**Children**						
1-3 years old	50²	13^*^	11	85.65/ 101.23	90.27/ 106.68	85.81/ 101.41
4-8 years old	70²	19^*^	15	82.05/ 103.93	86.47/ 109.53	82.20/ 104.11
**Women**						
19-30 years old	110^¹^	46^*^	38	53.25/ 64.47	56.12/ 67.94	53.35/ 64.58
31-50 years old	110^¹^	46^*^	38	53.25/ 64.47	56.12/ 67.94	53.35/ 64.58

* Institute of Medicine (IOM).

¹ TBCA

² Dietary Guidelines for Brazilian Children Under 2 Years Old, 2021.

## Discussion

This study characterized the nutritional composition of wild meat (mammals, birds, and reptiles) consumed in traditional food systems of the Brazilian Amazon, revealing similar proximate compositions across the different animal classes analyzed. The protein, lipid, and ash contents, as well as the energy composition of wild meat, were comparable to those found in conventional meats (chicken, beef, and pork), suggesting its potential as a high-quality nutritional source. These findings highlight the importance of wild meat as a viable and culturally significant nutritional resource, contributing to the food and nutritional security of traditional communities while promoting the preservation and appreciation of traditional dietary knowledge.

In general, we observed that the nutritional compositions of meat from different species show little variation, with more notable differences only in lipid content. This observation is supported by studies conducted, for example, in the Peruvian Amazon with similar species such as paca (*Cuniculus paca*), agouti (*Dasyprocta fuliginosa*), red brocket deer (*Mazama americana*), tapir (*Tapirus terrestris*), and collared peccary (*Pecari tajacu*), which also reported consistent protein and mineral levels [40]. However, the lipid content in our study was approximately three times higher than that observed in the Peruvian species, suggesting that environmental factors, such as food availability and seasonal variations, may significantly influence the lipid content of the meat [41].

This pattern of variation in lipid content, but not in protein levels, has also been observed in European studies. Species such as red deer (*Cervus elaphus*), wild boar (*Sus scrofa*), hare (*Lepus spp*.), wild rabbit (*Oryctolagus cuniculus*), and fallow deer (*Dama dama*) from the Mediterranean and Central European regions exhibited protein levels similar to our findings but considerably lower lipid levels [42]. In addition to the fact that distinct wildlife species were studied, this difference may be attributed to both methodological aspects—such as the specific selection of leaner cuts (loin and legs) in European studies—and environmental and behavioral factors of the species in different biomes. In a comparative meat study conducted in Germany [43], the use of infrared spectroscopy at multiple points of the carcasses revealed fat levels comparable to ours only in wild boars, differing from the results observed for roe deer. This suggests that intrinsic variations in the lipid composition of species, along with differences related to analytical methods and sample scope, may have influenced the findings.

In the comparative analysis between classes, no statistically significant differences were identified in protein content among mammals, birds, and reptiles. This result contrasts with some findings in the literature, such as a study conducted in the Uíge and Cuando-Cubango provinces of Angola, where reptiles exhibited significantly higher protein levels than birds [44]. However, Moro et al. [45] reported results more consistent with ours when analyzing different species: although the guinea fowl *(Numida meleagris* Linnaeus) showed higher protein levels than the yacare caiman (*Caiman crocodilus yacare* Dudin), this difference was not maintained when compared to the American alligator (*Alligator mississippiensis* Daudin), suggesting that variations may be more related to specific species than to animal classes.

Regarding ash content, which reflects the mineral composition, the results also showed no significant differences among classes. This contrasts with the findings of Oliveira et al. [30], who reported higher iron concentrations in bird meat compared to mammalian meat, suggesting a possible relationship between metabolic activity and mineral accumulation. The authors support the hypothesis that the more active metabolism of birds — particularly in muscle regions with higher activity, such as the thighs and breast — may contribute to this difference. However, more definitive conclusions about the mineral profile of these meats require targeted analyses using primary data and stricter control of variables that may influence composition, such as slaughter methods.

The comparative analysis of nutritional composition revealed a similarity between wild meats and conventional meats, such as chicken, beef, and pork. This nutritional convergence suggests that, in the absence of specific data on the composition of wild meat, information on domestic meats can serve as a reasonable approximation for macronutrient content. This finding is particularly relevant in the context of Food Matching, a tool that allows the estimation of the nutritional composition of a food based on similar foods [32]. This implies that, in the absence of precise data for all wild species, the use of domestic meat data may be a useful strategy for estimating the macronutrient nutritional value of these foods in the formulation of food policies and interventions, while more specific compositional data are obtained. The results of this study provide further scientific evidence supporting this potential.

Despite the general similarity observed in nutritional composition between wild and conventional meats, it is essential to recognize the need for additional studies to fully characterize the nutritional profiles of these meats, particularly regarding lipids. As previously discussed, fat content exhibited greater variability both among different species and within the same class. Furthermore, when comparing domestic and wild animals, some studies suggest that the physical activity level of the animal can significantly influence lipid composition, with wild animals tending to have a lower fat percentage due to their higher activity levels and energy expenditure in activities such as food foraging [46].

Additionally, our findings highlight the significant contribution of wild meats to the diet, particularly for vulnerable groups such as adult women and children. Considering the Recommended Dietary Allowances (RDA) and Estimated Average Requirements (EAR) for protein intake, the value of wild meat portions as an essential protein source for these populations becomes evident. The high protein and lipid content in wild meats underscores their role in meeting nutritional needs while also holding the potential to reduce pressure on grazing lands and promote forest preservation [47], in contexts of sustainable exploitation such as management [48], thereby contributing broadly to the promotion of food and nutritional security.

Thus, from a nutritional perspective, the consumption of wild meat emerges as a crucial strategy to mitigate deficiencies, especially given the high prevalence of anemia (59%) among riverine populations in the northern region [4]. The absence of wild meat consumption may exacerbate anemia risks [4]. Wild meat, rich in proteins and lipids, offers a vital alternative to combat protein malnutrition, which contributes to over 50% of infant deaths and clinical conditions such as kwashiorkor [49]. Protein malnutrition also significantly impacts women in preconception, pregnancy, and lactation periods, impairing child development and perpetuating food insecurity [50]. Considering the high prevalence of growth deficits in children under five years of age in riverine regions [51], wild meat consumption plays an important role in improving the health of these populations [4]. Fat, however, has been identified as a scarcer nutrient than protein in Amazonian indigenous diets [52], and hence the high fat content found in this study further supports the nutritional importance of wild meat for rural Amazonians.

The relationship between wild meat and food security in the context of Indigenous Peoples and Local Communities (IPLC) extends beyond nutrition to encompass cultural, economic, and conservation dimensions. The consumption of wild meat is deeply embedded in the dietary cultures of these peoples, serving a role beyond biological needs and representing a vital indicator of identity within social groups [23]. Studies from 15 countries show that imposing sanctions on wild meat consumption increases the risk of food insecurity, emphasizing its crucial role in traditional food systems and in rural African regions, where wild meat may account for about 20% of protein intake [53]. In this context, it is essential to adopt context-specific dietary practices that preserve regional identity without stigmatization or food prohibitions [54]. Health promotion in these communities should focus on strengthening their food culture and providing access to information about the safety and nutritional composition of their traditional foods [55]. Valuing wild-origin foods, rather than promoting dietary homogenization, fosters diversity and strengthens local identity [56].

However, the risks of defaunation—defined as the decline or extinction of animal populations due to human activities—must be acknowledged [57]. Defaunation can disrupt ecosystems by altering food chains and causing predator extinction [58]. In this context, sustainable hunting practices, including zoning and population control, present a promising solution. Zoning strategies used for species like *Arapaima gigas* (pirarucu fish) can be adapted for widely consumed species like paca (*Cuniculus paca*), ensuring population recovery and ecosystem balance [48]. Maintaining dietary diversity alongside conservation efforts aims to meet population needs while reducing environmental impacts, such as overfishing in aquatic ecosystems [59].

It is essential to emphasize that this study does not promote predatory hunting or uncontrolled commercialization of wild meat. Given that hunting is practiced in various contexts—whether as a hobby, for clothing, or as food [60] —it is crucial to institutionalize the issue for improved monitoring of biodiversity impacts [61]. Dependence on hunting for subsistence encompasses biological, sociocultural, and economic needs [61].

This study makes a significant contribution by assessing the feasibility of using data from conventional meats as a basis for estimating the nutritional composition of wild meat species in the absence of specific information. It also stands out for collecting and analyzing novel data on the composition of wild meat, substantially expanding knowledge about the diversity and quantity of samples by species. Furthermore, the methodologies employed are replicable, ensuring the reliability of the results and enabling their reproduction and use in future nutritional studies.

The study, however, faced some methodological limitations that merit attention. The primary limitation was the use of opportunistic sampling, both in terms of the individual organism and the specific cut of meat sampled. Opportunity sampling of animals was a deliberate choice aligned with ethical commitments to wildlife conservation, avoiding incentivizing the targeted hunting of animals for research purposes. While this approach reduces sampling efficiency, it reflects our responsibility towards environmental sustainability. Moreover, opportunistically sampling the cuts of meat that were donated to us prevented standardization for comparative analyses.

To improve future comparisons with conventional meats, the methodology will be refined to select species and cuts that are more appropriate for comparative purposes, including phylogenetic analysis. Additionally, the study was limited in evaluating the effect of the region of origin (flooded or non-flooded forests) on nutritional composition due to the low number of samples per area. Future studies that consider these variables and include larger sample sizes—while respecting conservation principles and leveraging existing consumption practices—will be crucial for a deeper understanding of the nutritional characteristics of wild meats.

## Conclusion

The wild meats, which are widely consumed in traditional food systems in the Brazilian Amazon, have a nutritional value equivalent to conventional meats, considering macronutrients. This reinforces their potential as a sustainable and culturally relevant food alternative, serving as an important path in promoting food and nutritional security for riverside peoples and communities. It is evident that in the absence of nutritional information about wild meats, it is possible to equate them with conventional meats in terms of macronutrient composition. The study presents an innovative contribution by providing concrete data on the nutritional composition of these meats, which are often neglected. Moreover, the study emphasizes the importance of integrating traditional food practices in the promotion of food and nutritional security, valuing local knowledge, and expanding discussions on sustainability, biodiversity, and cultural conservation within the context of traditional communities.

## Supporting information

S1 Checklist Inclusivity in global research questionnaire. (DOCX)

S2 File Results of the statistical analysis. (DOCX)

S3 File Database. (XLSX)
