# Supplementary material for: Proximate composition of wild meats present in traditional food systems of the Brazilian Amazon
Source: PLoS One. 2025 Jul 21;20(7):e0327783. doi: 10.1371/journal.pone.0327783 (PMC12279099; doi:10.1371/journal.pone.0327783)
Supplement: S1 Checklist — (DOCX) [file pone.0327783.s001.docx]

Inclusivity in global research

PLOS’ policy on inclusivity in global research aims to improve transparency in the reporting of research performed outside of researchers’ own country or community and ensures that PLOS publications reporting global research adhere to high standards for research ethics and authorship. Authors of relevant research articles may be asked to complete the questionnaire below, which outlines ethical, cultural, and scientific considerations specific to inclusivity in global research. This questionnaire may be requested when researchers have travelled to a different country to conduct research, if research uses samples collected in another country, research with Indigenous populations or their lands, or if research is on cultural artefacts. Researchers travelling to another country solely to use laboratory equipment will not normally be required to complete the questionnaire. However, the questionnaire can be requested at the journal’s discretion for any submission – if you have been requested to complete this questionnaire by the PLOS journal you submitted to, please do so.

Please complete the questionnaire below and include this as a Supporting Information file with your manuscript. Note that if your paper is accepted for publication, this checklist will be published with your article in the supporting information files. Please ensure that you reference the checklist in the main body of your manuscript. We suggest adding a subsection ‘Inclusivity in global research’ to your Methods section and adding the following sentence: “Additional information regarding the ethical, cultural, and scientific considerations specific to inclusivity in global research is included in the Supporting Information (SX Checklist)”

The questions have been designed to be applicable to a wide range of study types, and there are subsections for both human subjects research and non-human subjects research. If any of the questions are not relevant to your research please mark them as “N/A” as appropriate.

**Ethical considerations, permits and authorship**

*This section is applicable to all research types.*

Provide details as to who granted permissions and/or consent for the study to take place in the Methods section of your manuscript. This should include the names of **all** ethics boards, governmental organizations, community leaders or other bodies that provided approval for the study. If individuals provided approval refer to these people by their role or title but do not list their name(s).


If there were any deviations from the study protocol after approval was obtained please provide details of these changes in the Methods section of your manuscript.
Did this study involve local collaborators that are residents of the country where the research was conducted or members of the community studied? If you do not have any authors from said communities, please provide an explanation for this below.



Everyone listed as an author should meet PLOS’ criteria for authorship and all individuals who meet these criteria should be included in the author byline, rather than the acknowledgements. For further information please see the journal’s Authorship Policy.

**Human subjects research (e.g. health research, medical research, cross-cultural psychology)**

Did you obtain written informed consent from a representative of the local community or region before the research took place? How did you establish who speaks for the community? Details of written informed consent obtained from study participants should be reported separately in the Methods section of your manuscript.

The Mamirauá Sustainable Development Institute’s Research Ethics Committee (CEP) agreed that written permission for collecting samples of wildmeat samples could cause discomfort to community members, particularly when they were the hunter. As such our ethics permission (CAAE: 55899222.7.0000.8117) states that we require oral permission from community members to collect animal tissue samples. Moreover, written permission was impossible given that we maintain anonymity by never noting the identity of community members that donate the sample. Written informed consent was further considered inappropriate given that much of the population is illiterate.

How did members of the local community provide input on the aims of the research investigation, its methodology, and its anticipated outcome(s)?

Yes, members of the riverside communities contributed to this research by generously providing samples.

When engaging with the local community, how did you ensure that the informed consent documents and other materials could be understood by local stakeholders?

Community consent was obtained orally. All communities involved in this study are considered long-term collaborators of the Mamirauá Institute, with a prior understanding of scientific research. In this study, Mamirauá Institute researcher (Daniel Tregidgo) initially approached a leader of each community, referred to as a president. Following discussion of the objectives and methodology of the study, and permission granted by the president, it was common that a community meeting was called, with all community members invited. During this meeting, Dr Tregidgo would again explain the study in a way understood by community members, based on his 18 years experience working with Amazonian riverside communities. The floor was then opened to questions, before the community agreed to accept the research.

A summary of basic information about the research, which followed the layout of brazilian informed consent documents (TCLE) with objectives, methods, risks and discomforts, benefits, anonymity, compensation and contacts, was presented to community leaders, and offered to community members that donated samples. This document was written in simple Brazilian Portuguese, and approved by the Mamirauá Sustainable Development Institute’s Research Ethics Committee (CEP). However, given the reasons already stated in this questionnaire, both CEP and the researchers consider this a secondary means of communicating the study aims and obtaining consent, which was primarily undertaken orally.

Will the findings of the research be made available in an understandable format to stakeholders in the community where the study was conducted (e.g. via a presentation, summary report, copies of publications, etc.)? Please provide details of how this will be achieved.

Yes, we intend to prepare materials and present them in workshops for the community. The research team, namel DT, JM and MJ, have a strong reputation in dissemination and education regarding food and nutritional security in all of the studied communities, have undertaken numerous workshops and presented educational materials to them over the past few years. We also work in close collaboration with local environmental and health organisations and stakeholders. Given our experience and reputation, we are confident that our findings will be understood and well received.

**Non-human subjects research using specimens/ animals collected as part of the study, or those housed in archival collections. Examples include archaeology, paleontology, botany and zoology.**

Did the permission you obtained from a local authority to perform the study include an agreement on access to outputs and benefit sharing? This may include procedures to enable fair distribution of the benefits and resources arising from the research performed. Please include any details of Prior Informed Consent and Benefit Sharing Agreements obtained. These may be required by field-specific regulations, for example the Convention on Biological Diversity (CBD) and the associated Nagoya Protocol.

N/a

If the material used in your study was imported, please A) provide the year it was imported and B) indicate whether permits were obtained to import/export the materials used, C) provide details of any permits obtained. If this information is not available, please indicate this.

N/a

If you used archival specimens, please state how the material used in your study was acquired by the institute it is held in and provide details of any permits obtained for the original excavations/ sample collection. If this information is not available, please indicate this.

N/a

How was the potential cultural significance of the materials collected in your study to local communities considered in your research design? Were Indigenous peoples and/or local researchers and institutions involved with archaeological excavations / collection of specimens? If so, please provide a description of their involvement.

Sample collection was led by co-author Daniel Tregidgo, from the local research institution, the Mamirauá Institute. Local riverside communities donated small samples of muscle and liver from hunted animals. Wildmeat is known to be central to the diet of these local people, and this study was designed to understand what that means in nutritional terms.

If your manuscript includes photographs of human remains please indicate whether authors obtained permission from descendants or affiliated cultural communities to do so.

N/a
