# Supplementary material for: Proximate composition of wild meats present in traditional food systems of the Brazilian Amazon
Source: PLoS One. 2025 Jul 21;20(7):e0327783. doi: 10.1371/journal.pone.0327783 (PMC12279099; doi:10.1371/journal.pone.0327783)
Supplement: S2 File — (DOCX) [file pone.0327783.s002.docx]

**Supplementary Material 1.** Results of the Statistical Analyses.

| **Nutrient** | **Results** |
| --- | --- |
| Protein (g) | Shapiro-Wilk: p-value = 0.01525  Kruskal-Wallis: p-value = 0.3055 |
| Lipids (g) | Shapiro-Wilk: p-value = 0.0000004358  Kruskal-Wallis: p-value = 0.07164 |
| Ashes (g) | Shapiro-Wilk: p-value = 0.01239  Kruskal-Wallis: p-value = p-value = 0.13 |

The p-value was considered statistically significant when p < 0.05.
